# The Function of Heterodimeric AP-1 Comprised of c-Jun and c-Fos in Activin Mediated Spemann Organizer Gene Expression

**DOI:** 10.1371/journal.pone.0021796

**Published:** 2011-07-29

**Authors:** Sung-Young Lee, Jaeho Yoon, Hyun-Shik Lee, Yoo-Seok Hwang, Sang-Wook Cha, Chul-Ho Jeong, Jong-Il Kim, Jae-Bong Park, Jae-Yong Lee, SungChan Kim, Mae Ja Park, Zigang Dong, Jaebong Kim

**Affiliations:** 1 Department of Biochemistry, College of Medicine, Hallym University, ChunCheon, Kangwon-Do, Republic of Korea; 2 The Hormel Institute, University of Minnesota, Austin, Minnesota, United States of America; 3 Department of Anatomy, School of Medicine, Kyungpook National University, Daegu, Republic of Korea; 4 School of Life Sciences, College of Natural Sciences, Kyungpook National University, Daegu, Republic of Korea; 5 College of Pharmacy, Keimyung University, Daegu, Republic of Korea; National University of Singapore, Singapore

## Abstract

**Background:**

Activator protein-1 (AP-1) is a mediator of BMP or FGF signaling during *Xenopus* embryogenesis. However, specific role of AP-1 in activin signaling has not been elucidated during vertebrate development.

**Methodology/Principal Findings:**

We provide new evidence showing that overexpression of heterodimeric AP-1 comprised of c-*jun* and c-*fos* (AP-1^c-Jun/c-Fos^) induces the expression of BMP-antagonizing organizer genes (*noggin, chordin and goosecoid*) that were normally expressed by high dose of activin. AP-1^c-Jun/c-Fos^ enhanced the promoter activities of organizer genes but reduced that of *PV.1*, a BMP4-response gene. A loss of function study clearly demonstrated that AP-1^c-Jun/c-Fos^ is required for the activin-induced organizer and neural gene expression. Moreover, physical interaction of AP-1^c-Jun/c-Fos^ and Smad3 cooperatively enhanced the transcriptional activity of *goosecoid* via direct binding on this promoter. Interestingly, Smad3 mutants at c-Jun binding site failed in regulation of organizer genes, indicating that these physical interactions are specifically necessary for the expression of organizer genes.

**Conclusions/Significance:**

AP-1^c-Jun/c-Fos^ plays a specific role in organizer gene expression in downstream of activin signal during early *Xenopus* embryogenesis.

## Introduction

AP-1 (activator protein-1) protein is an evolutionarily conserved bZip family transcriptional factor composed of Jun family members (c-Jun, JunB and JunD) and Fos family members (c-Fos, FosB, Fra-1 and Fra-2). Jun proteins form homodimers of Jun family members or heterodimers with Fos proteins, whereas Fos proteins do not form homodimers and require Jun proteins to bind DNA [1]. Each of AP-1 components is differentially expressed and regulated with subtly different functions [2]. Moreover, diverse combinations of AP-1 components are known to mediate various biological processes such as cell proliferation, differentiation, and development [2], [3], [4], [5], [6]. However, despite increasing knowledge regarding the physiological functions of AP-1, more specific role and target genes of AP-1 components still remains to be investigated.

During early vertebrate development, the mesoderm-inducing process is one of the most important events. Several mesoderm-inducing factors have been identified using amphibian embryo systems [7], [8], [9]. In *Xenopus* embryos, normal mesoderm formation largely depends on FGF [10], [11], [12] and activin signaling [13]. FGF and activin can induce a wide variety of mesodermal genes such as *Xbra* [14], *Xhox3* [15], *Xwnt8* [16], and *Xnot* [17]. However, FGF and activin have important differences in terms of mesoderm induction. Analysis of the inducing activities of activin and FGF revealed that at high concentration of activin tends to induce dorsal and axial structures such as notochord and muscle, whereas FGF induces ventral mesoderm, such as mesenchyme, mesothelium and lateral mesoderm [18]. Moreover, in animal cap explants, relatively high concentration of activin has been found to induce dorsal mesoderm markers (Spemann organizer genes; *chordin, noggin*, and *goosecoid*) and some neural markers (*N-CAM, otx2*, and *zic3*), while all concentrations of fibroblast growth factors (bFGF or eFGF) failed to induce dorsal mesoderm and neural markers [18], [19], [20]. Regarding mesoderm formation by FGF signaling, we previously reported that AP-1 is an essential component of the mesoderm maintenance machinery, which is mediated by autocatalytic loop of eFGF/Ras/Xbra in animal cap explants of *Xenopus* embryos [21], [22]. Additionally, we reported the involvement of AP-1 in BMP-4 signaling and its expression [23], [24]. However, physiological function of AP-1 in activin-induced cell differentiation processes has not been elucidated during *Xenopus* development.

In this paper, we provide new insight into the function of heterodimeric AP-1 comprised of c-*jun* and c-*fos* (AP-1^c-Jun/c-Fos^) in activin-mediated organizer gene expression. Importantly, physical interaction of AP-1 and Smad3 is necessary for the induction of organizer genes including *noggin*, *chordin* and *goosecoid*, but not for that of *Xbra*. Taken together, we suggest that AP-1^c-Jun/c-Fos^ has an important role in activin-mediated expression of BMP-antagonizing organizer genes.

## Materials and Methods

### Ethics Statement

Approval from the Institutional Animal Care and Use Committee (IACUC) is not required for the experimental use of amphibian and reptiles in Korea. All members of our research group members attended educational and training courses for the appropriate use of experimental animals.

### Embryo injection and animal cap assay

*Xenopus laevis* embryos were obtained by artificial fertilization [25]. Vitelline membranes were removed by immersing embryos in a 2% cysteine solution (pH 8). Embryos at the one- or two-cell stage were injected in the animal pole with messenger RNA as described in the Figure Legends. Animal caps, the area around the animal (pigmented) pole of the blastula embryos, were dissected from the injected embryos at stage 8–9 and cultured to various stages in 67% Leibovitzs L-15 medium (Invitrogen, Carlsbad, CA) containing BSA (1 mg/ml), 7 mM Tris-HCl (pH 7.5), and gentamicin (50 µg/ml) for 1 or 2 days. Activin (Sigma, St. Louis, MO) was added to L-15 medium [24], [26], [27]. The isolation and differentiation of animal cap cells, the so-called animal cap assay, is a useful method for investigating the mechanism of cell differentiation at the molecular level. The cells of the animal caps retain pluripotentiality and upon exposure to specific inducers, the animal cap can differentiate into neural, mesodermal, or endodermal tissues, equivalent to mammalian embryonic stem cells.

### *In vitro* transcription

All synthetic mRNAs used for microinjection were produced by *in vitro* transcription. Each of the cDNAs were linearized and used for the *in vitro* synthesis of capped mRNAs using an *in vitro* transcription kit (Ambion, Austin, TX) in accordance with the manufacture's instructions. Synthetic RNA was quantified by ethidium bromide staining and compared with a standard RNA.

### RNA isolation and reverse transcription-polymerase chain reaction (RT-PCR)

Total RNA was extracted from whole embryos or cultured explants using TRIzol reagent (Tel-Test, Inc., Friendswood, TX) by following the manufacturer's instructions. RT-PCR was performed with a Superscript pre-amplification system (Invitrogen, Carlsbad, CA). PCRs were performed using the following conditions; 94°C for 5 minutes, 19–28 amplification cycles (94°C for 1 min, the appropriate annealing temperature for 1 min, and 72°C for 1 min) as described by the *Xenopus* Molecular Marker Resource (XMMR; University of Texas), and a final extension at 72°C for 10 min.

### Luciferase assay

Both of luciferase reporter plasmid DNA and β-galactosidase reporter gene as a internal control were injected alone or together with the indicated mRNA into one- or two-cell stage embryo as described in the figure legends. After injection, the animal caps were excised from embryos (stage 8.5–9) and cultured until indicated stage as described in the figure legends. Luciferase activities were measured using a luciferase assay system according to manufacturer's instructions (Promega, Madison, WI), and was normalized against the *β–galactosidase* activity. Five or four groups of animal caps, four to five animal caps per group, were pooled and homogenized in 10 µl of lysis buffer per animal cap. All experiments were repeated at least three times using independent samples.

### Plasmid construct

The *Xenopus c-jun* (GeneBank Accession Number; AJ243955) and *c-fos* (GeneBank Accession Number; BC079689) cDNA was isolated from *Xenopus* cDNA library and were inserted into EcoRI and XbaI sites of pCS2 (+) vector and flag-tagged pCS2 (+) vector by PCR. The rat *c-jun* and *c-fos* cDNA were obtained as described [22] and were inserted into flag-tagged pCS2 (+) vector by PCR. Smad3 and 6myc-Smad3 were inserted into pCS2 (+) vector. The mutation of Smad3 (K40, 41, 43, 44A) was generated using the QuickChange II site directed mutagenesis kit (Stratagene, Cedar Creek, TX).

The luciferase reporter genes containing the promoter regions of *noggin*, *goosecoid*, and *PV.1* were used for checking responses to AP-1 or activin signaling. The 2066 bp 5′ flanking region of *noggin* was cloned into KpnI/BglII-treated pGL3 basic vector [28]. The 240 bp 5′ flanking region of *goosecoid*, amplified by PCR, was inserted into KpnI/HindIII-digested pGL3 promoter vector (−240 gsc/Luc). This −240 bp is including the minimum promoter containing the essential region for the high-level transcription of *goosecoid*. These reporter genes, which contain promoter sequences, are from activin responsive molecules which are activated by activin or the mediators of activin signaling, Smad2 and Smad3 [29]. On the other hand, the 2.5 kb 5′ flanking region of *PV1*, which is down-regulated by activin, was inserted into XhoI/HindIII-digested pGL2 basic vector.

### Morpholino oligos

The antisense morpholino oligonucleotides (MOs) were obtained from Gene Tools (Philomath, OR). The MO sequences were as follows: MO-Jun54 5′-CTGGAGCTTATGTCAGTGTGA-3′; MO-Jun55 5′-GTAGTTTCCATCTTTGCGTTCATAC-3′; Cont-MO 5′-CCTCTTACCTCAGTTACAATTTATA-3′. MO-Jun54 and 55 were designed to bind to complementary sequences found in two kinds of *Xenopus c-jun* mRNAs [30], and prevent the translations of these *c-jun* mRNAs. Morpholino oligos riboside moieties are substituted with nitrogen-containing morpholine moieties and are phosphorodiamidate linked [31]. Oligos were re-suspended in sterile water and injected in dose of 20 ng per embryo.

### Chromatin immunoprecipitation (ChIP)

ChIP was performed as previously described [32], with the following modifications. For ChIP analysis, embryos at the one cell stage or two cell stages were injected in the animal pole with 1 ng of messenger RNA, as described in the figure legends. About 100–150 of injected embryos were fixed in 1.85% formaldehyde in 0.1 X MBS for 30 min at room temperature. Mouse monoclonal anti-Flag (Sigma, St. Louis, MO), anti-Myc (Santa Cruz, Santa Cruz, CA) and rabbit polyclonal anti-c-Jun (Santa Cruz, Santa Cruz, CA) were used for immunoprecipitation. Chromatin solution was pre-cleared with Protein A/G PLUS-Agarose beads (Santa Cruz, Santa Cruz, California). Embryos were homogenized using a VC 50T (Sonics & Materials Inc.), 20–25 times for 10 sec at amplitude 40. *Goosecoid* promoter was assayed by immunoprecipitated DNA using the promoter primers (forward) 5′- CCGTTAATG-TCCCATCAC-3′ (position -240) and (reverse) 5′-TGTGTGTGC GTCTCTCGCT-3′ (position +50).

### Immunoprecipitation and western blot analysis

Embryos were injected at the one cell stage with RNA constructs as described, and frozen at stage 10.5. They were then homogenized in lysis buffer (50 mM Tris [pH 7.4]), 150 mM NaCl, 1% NP-40, 0.25% sodium deoxycholate, 0.1% SDS, 50 mM NaF and 1 mM Na_3_VO_4_) containing of 1 mM PSMF, 15 mM β-glycerophosphate, 1 X proteinase inhibitor cocktail (Calbiochem, Darmstadt, Germany). Cell lysates were cleared by centrifugation, and precipitations were performed by incubating overnight with mouse monoclonal anti-Flag (Sigma, St. Louis, MO), anti-Myc (Santa Cruz, Santa Cruz, CA) or rabbit polyclonal anti-c-Fos (Santa Cruz, Santa Cruz, CA), then add Protein A/G Plus Agarose (Santa Cruz, Santa Cruz, CA) and incubated for 3 h at 4°C. Unbound proteins were removed by washing four times with lysis buffer. Bound proteins were harvested by boiling in sample buffer, and resolved by electrophoresis in 10% SDS-polyacrylamide gels. Myc-tagged, Flag-tagged proteins and AP-1 proteins were visualized after western blotting using rabbit polyclonal anti-Myc (Santa Cruz, Santa Cruz, CA), mouse monoclonal anti-Flag, rabbit polyclonal anti-c-Jun (Cell Signaling, Danvers, MA) and rabbit polyclonal anti-c-Fos. Proteins were visualized using ECL Western blotting detection reagents (Amersham, Pittsburgh, PA).

### Whole mount *in situ* hybridization

Embryos were injected with indicated mRNAs, and then processed for whole-mount in situ hybridization by using standard methods [33] with following probes: *goosecoid*, *chordin*, *Xbra* and *Xvent2*.

## Results

### AP-1^c-Jun/c-Fos^ induces Spemann organizer genes, similarly to activin signaling in animal cap explants

**Figure 1:**
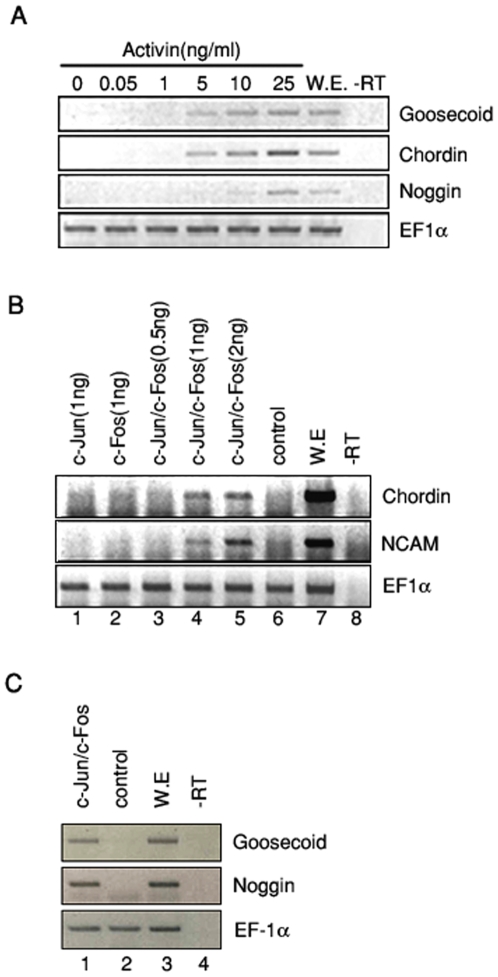
AP-1 functions similar to activin in dorsal mesoderm and neural gene expression in animal cap explants. (A–C) RT-PCR analysis of organizer genes (*chordin, noggin* and *goosecoid*) or late neural specific marker (*N-CAM*) in activin treated animal cap explants or in mRNAs encoding *c-jun* or/and *c-fos* injected animal cap explants. (C) Animal caps isolated from embryos injected with 1 ng of mRNAs encoding *c-jun* and *c-fos* was used for RT-RCR analysis. EF-1α, a loading control; -rt, control reaction without reverse transcriptase; cont, animal cap samples obtained from non-injected embryos; we, whole embryo as a positive control.

To determine the function of heterodimeric AP-1 comprised of c-*jun* and c-*fos* (AP-1^c-Jun/c-Fos^) in activin-mediated organizer gene expression. The gene expression profiles induced by AP-1^c-Jun/c-Fos^ and activin signaling were compared. We first confirmed the expression of Spemann organizer genes by activin using animal cap assay. Animal caps were incubated with activin and expression of Spemann organizer genes (*goosecoid, chordin*, and *noggin*) were examined by RT-PCR. As expected, activin induced the organizer genes of the early dorsal mesoderm markers in a dose-dependent manner (Fig. 1A). To investigate the induction of Spemann organizer genes by AP-1, synthetic mRNAs encoding AP-1 (c-*jun* and c-*fos*) were injected into the animal poles of fertilized eggs. Excised animal caps were used for RT-PCR analysis. Co-injection of *c-jun* and *c-fos* also induced the expression of Spemann organizer genes as well as *N-CAM*, which is pan-neural markers and expressed only by high dose of activin, in a dose-dependent manner (Figs. 1B & C). On the other hand, the expression of epidermis marker (*XK81*) and ventral marker (*PV.1*), which has been known to be a BMP-4 responsive gene and to be inhibited by activin signaling, was inhibited by AP-1^c-Jun/c-Fos^ in a dose dependent manner (Supplementary Figs. S1A & B). Importantly, injection of *c-jun* or *c-fos* alone, or a low dosage of AP-1^c-Jun/c-Fos^ (0.5 ng) did not induce the expression of those genes in animal cap explants (Fig. 1B, lane 1–3), implying that enough amount of heterodimeric AP-1^c-Jun/c-Fos^ acts functionally. Therefore, these results demonstrate that AP-1^c-Jun/c-Fos^ functions similar to activin as an inducer of BMP-antagonizing organizer-specific genes in animal cap explants.

### AP-1^c-Jun/c-Fos^ enhances the promoter activities of activin-responsive organizer genes

**Figure 2:**
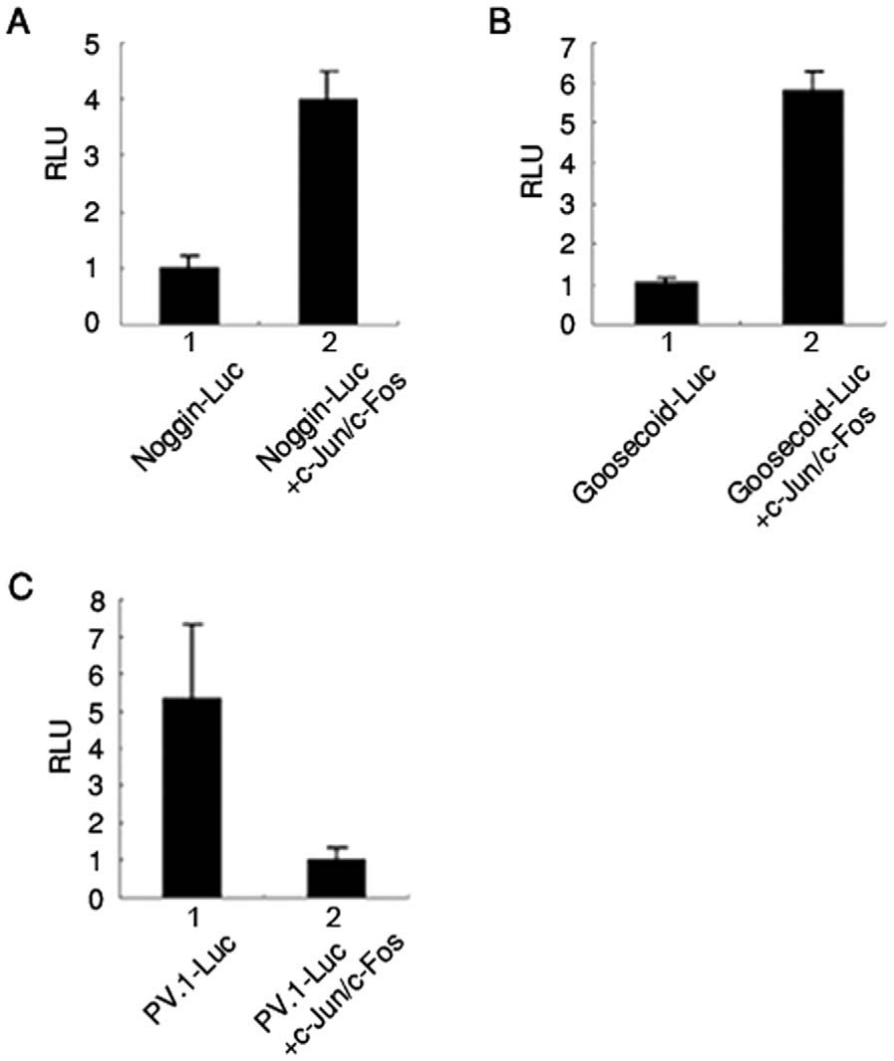
AP-1 enhances promoter activity of activin response genes, but reduces that of BMP4-response gene. (A–C) Luciferase assay using animal caps (at stage 10.5 equivalent). Indicated mRNAs or expression plasmids were used: lane 1, *Noggin*, *Goosecoid*, or *PV.1* promoter luciferase reporter gene; lane 2, co-injection with 1 ng of mRNAs encoding AP-1 (*c-jun* and *c-fos*). All values are averages of at least three independent experiments. RLU, Relative luciferase activity.

To examine whether AP-1^c-Jun/c-Fos^ up-regulates promoter activities of activin-responsive organizer genes, luciferase constructs which contains promoters of *noggin* and *goosecoid* genes were tested. Consistent with RT-PCR results shown in Figs. 1, the reporter activities were increased 3 to 6 folds by co-injection of *c-jun* and *c-fos* mRNAs (Figs. 2A & B). However, injections of *c-jun* or *c-fos* mRNA alone or other AP-1 components (JunD/c-Fos or JunD/FosB) did not increase *goosecoid* or *noggin* promoter activities (data not shown). Moreover, the reporter activity of *PV.1* promoter, which has been known to be a BMP-4 responsive gene and to be inhibited by activin signaling, was significantly decreased by injection of AP-1^c-Jun/c-Fos^ (Fig. 2C). These results indicate that heterodimeric AP-1^c-Jun/c-Fos^ up-regulates transcription of activin-responsive organizer genes but not that of a BMP-responsive gene (*PV.1*).

### AP-1 is required for the expression of organizer genes mediated by activin signaling

**Figure 3:**
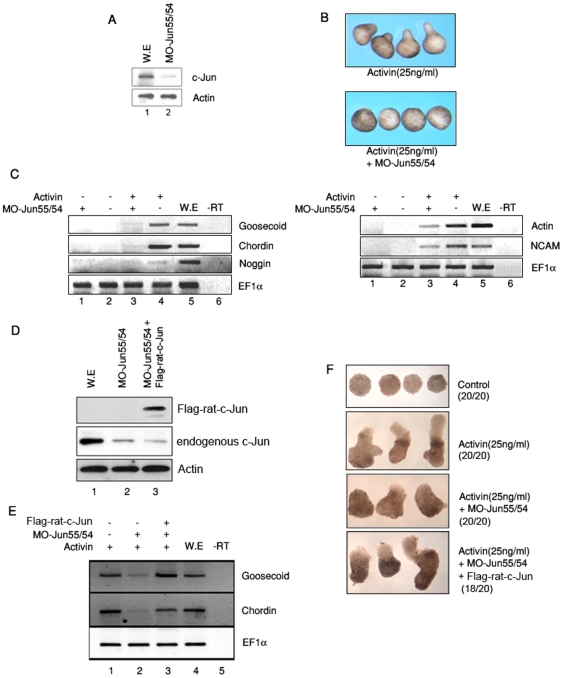
AP-1 is required for activin signaling. (A) MO-Jun55/54 (20 ng of MO-jun55 and 54) specifically inhibit the translation of endogenous c-Jun. (B–C) Animal caps isolated from embryos injected with or without MO-Jun55/54 were cultured until stage 10.5 or stage 24. (B) The morpholino oligonucleotide Juns (MO-Jun55/54) inhibited morphological change induced by activin. (C) RT-PCR analysis of animal caps injected with the indicated MO-Jun55/54 or treated with activin (25 ng/ml). MO-Jun55/54 suppressed the markers induced by activin signaling (lane 3 and 4). (D–F) Animal caps isolated from embryos injected with or without MO-Jun55/54 and mRNA of Flag rat c-Jun were cultured in presence of activin until stage 10.5. (D) Inhibition of endogenous c-Jun and Flag rat c-Jun translation were confirmed by western blotting. (E) RT-PCR analysis of animal caps. Co-injection of rat c-Jun rescued the organizer genes suppressed by MO-Jun55/54. (F) The phenotype of animal caps inhibited by MO-Jun55/54 was rescued by co-injection of rat c-Jun mRNA. 20 embryos that had received a microinjection of indicated mRNA or MOs were used for animal cap isolation at the blastula stage. We presented the number of animal cap's phenotype for each group. EF-1α, a loading control; -rt, control reaction without reverse transcriptase; cont, animal caps sample dissected from non-injected embryos; we, whole embryo as a positive control; organizer genes, *chordin*, *noggin* and *goosecoid*: later dorsal mesoderm marker, *Actin*; neural marker, *N-CAM*.

To investigate the physiological role of AP-1 in *X. laevis* development, we performed a loss-of-function study using morpholino antisense directed against c-Jun. We generated two antisense morpholino oligonucleotides (MO-Jun54 and MO-Jun55) which were designed to block translation of two forms of c-Jun as described in Materials and Methods. We confirmed that MO-Jun54 and MO-Jun55 (MO-Jun 54/55) effectively inhibited translation of c-Jun as revealed by western blotting (Fig. 3A). Cont-MO did not affect translation of c-Jun (Supplementary Fig. S2A). Consistently, depletion of c-Jun inhibited the elongation of animal cap explants induced by activin, implying that c-Jun might act as a downstream molecule of activin signaling (Fig. 3B).

Based on our findings that AP-1^c-Jun/c-Fos^ functions similar to as an inducer of organizer-specific genes and activin stimulates AP-1 reporter activity in animal cap explants (data not shown), we then examined whether depletion of c-Jun can inhibit the expression of activin-induced organizers and neural marker by RT-PCR analysis. Notably, MO-Jun54/55 effectively inhibited the expression of activin-induced organizer genes and neural marker including later dorsal mesoderm marker (Fig. 3C). Cont-MO did not affect activin-induced organizer genes (Supplementary Fig. S2B). We then investigated whether these suppressed expression of organizer genes, as well as the defective phenotype of animal caps induced by MO-Jun55/54 could be rescued by overexpression of flag-rat *c-jun* mRNA. Overexpression of rat c-Jun and depletion of endogenous c-Jun was confirmed by Western blot (Fig. 3D). We also verified that overexpression of rat *c-jun* mRNA under depletion of endogenous c-Jun effectively rescued the suppressed expression of organizer genes (Fig. 3E) and defective phenotype of animal caps (Fig. 3F).

Therefore, these results clearly excluded the possibility of artifact in MO-55/54 knock-down experiment and confirmed the genuine roles of AP-1 as a downstream mediator of activin signaling in *Xenopus* development.

**Figure 4:**
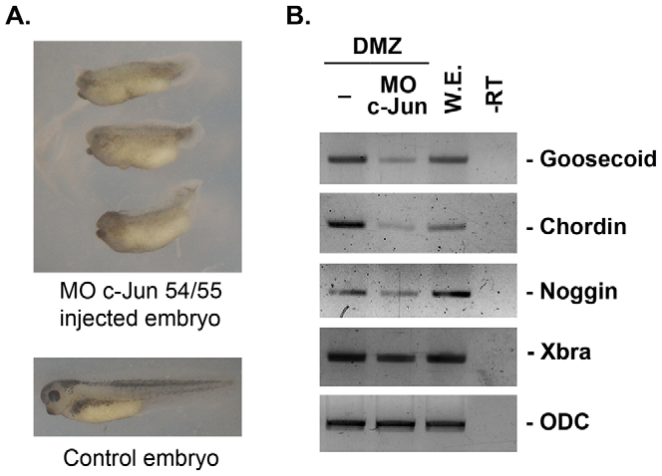
Physiological role of AP-1 in whole embryo. (A) Embryos were injected with 20 ng of MO-Jun54/55 into animal regions of one- or two-cell stage embryos and cultured until stage 40. c-Jun depleted embryos showed short axis and disrupted anterior structure. (B) Marginal zone of two dorsal blastomeres (DMZ) of four-cell stage embryos injected with 20 ng of MO-Jun54/55 was isolated and cultured until stage 11. Spemann organizer genes (*noggin, goosdcoid* and *chordin*) and mesoderm marker (*Xbra*) were investigated by RT-PCR analysis. The organizer genes expressed in DMZ were significantly reduced by depletion of c-Jun. ODC, a loading control; -RT, control reaction without reverse transcriptase; W.E., whole embryo as a positive control.

Additionally, we tested the phenotypes of c-Jun depleted embryos to investigate the physiological role of AP-1 in whole embryos. Injection of MO-Jun54/55 into the animal region of embryos at the one- or two-cell stage caused shortened dorso-anterior axis and disrupted anterior structure indicating that depletion of AP-1 in whole embryos caused the disruption of dorsal structure (Fig. 4A). Cont-MO-injected embryos showed similar phenotype of uninjected control embryos (data not shown). In order to further investigate the effect of MO-Jun 54/55 in embryos, MO-Jun 54/55 was injected into the marginal zone of two dorsal blastomeres (DMZ) of four-cell stage embryos and the isolated DMZ explants were used for RT-PCR analysis. RT-PCR results showed a significantly reduced expression of Spemann organizer genes (*chordin*, *noggin*, and *goosecoid*) in the c-Jun-depleted DMZ explants. Otherwise pan-mesoderm marker, *Xbra* was not significantly repressed by depletion of c-Jun (Fig. 4B).

Taken together, our results demonstrate that AP-1 is necessary for the regulation of organizer genes in DMZ region and whole embryos as well as animal cap explants induced by activin signaling.

### AP-1^c-Jun/c-Fos^ is involved in Smad3-mediated organizer gene expression

**Figure 5:**
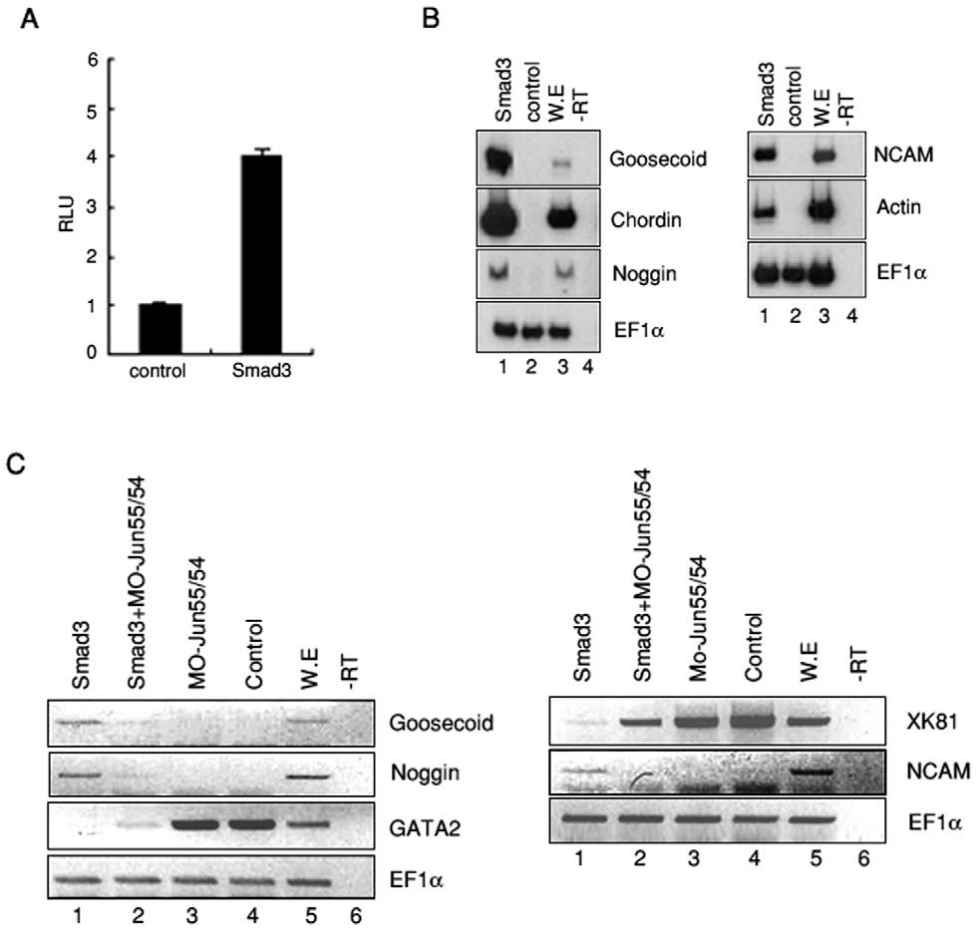
AP-1^c-Jun/c-Fos^ is involved in Smad3-mediated organizer gene expression. (A) (AP-1)4-luciferase assay using animal cap explants injected with Smad3 mRNA. Smad3 enhanced the AP-1 activity. (B–C) RT-PCR analysis of animal caps expressing the indicated mRNA of Smad3 or MO-Jun55/54. (B) Smad3 induced organizer genes (*goosecoid, chordin* and *noggin*), later dorsal mesoderm marker (*Actin*) and neural marker (*NCAM*). (C) MO-Jun55/54 suppressed Smad3-induced organizer genes (*goosecoid* and*noggin*) at stage 10.5 and neural marker (*NCAM*) at stage 24. On the other hand, ventral mesoderm marker (*GATA2*) and non-neural marker (*XK81*) were increased by co-injection of MO-Jun55/54 and Smad3 mRNA. EF-1α, a loading control; -rt, control reaction without reverse transcriptase; cont, animal caps sample dissected from non-injected embryos; we, whole embryo as a positive control.

Smad2 and Smad3 are representative transcription factors that activate gene expression in response to TGFβ/activin signaling pathway. These closely related proteins Smad2 and Smad3 were known to play different roles in early mice development [34]. Recently, the different role of Smad2 and Smad3 regarding particular gene regulation was reported during *Xenopus* development [35]. Of note, several lines of evidences describing a functional cooperatives of between Smad3 and AP-1 in TGFβ signaling [36] and a crucial role of Smad3 in the regulation of *chordin* [35] prompted us to investigate the roles of Smad3 in activin-mediated organizer gene regulation. In this regard, we examined whether AP-1 luciferase activity might be regulated by Smad3, a crucial mediator of activin signaling. Consistent with activin, Smad3 significantly induced AP-1 activity in animal cap explants (Fig. 5A). We also confirmed that Smad3 induces the expression of Spemann organizer genes (*goosecoid*, *chordin*, and *noggin*) as well as late dorsal mesoderm marker (*Actin*) and neural marker (*N-CAM*) in animal cap explants (Fig. 5B).

Importantly, co-injection of MO-Jun54/55 with Smad3 effectively inhibited the expression of organizer genes and neural marker induced by Smad3 (Fig. 5C), while the expression of ventral (*GATA-2*) and epidermis marker (*XK81*), which were inhibited by Smad3, were increased by MO-Jun55/54 (Fig. 5C). Therefore, these results indicate that the expression of organizer genes by Smad3 requires AP-1^c-Jun/c-Fos^ during *Xenopus* development.

### AP-1^c-Jun/c-Fos^ and Smad3 cooperatively regulate the transcription of goosecoid gene through direct binding to its promoter

**Figure 6:**
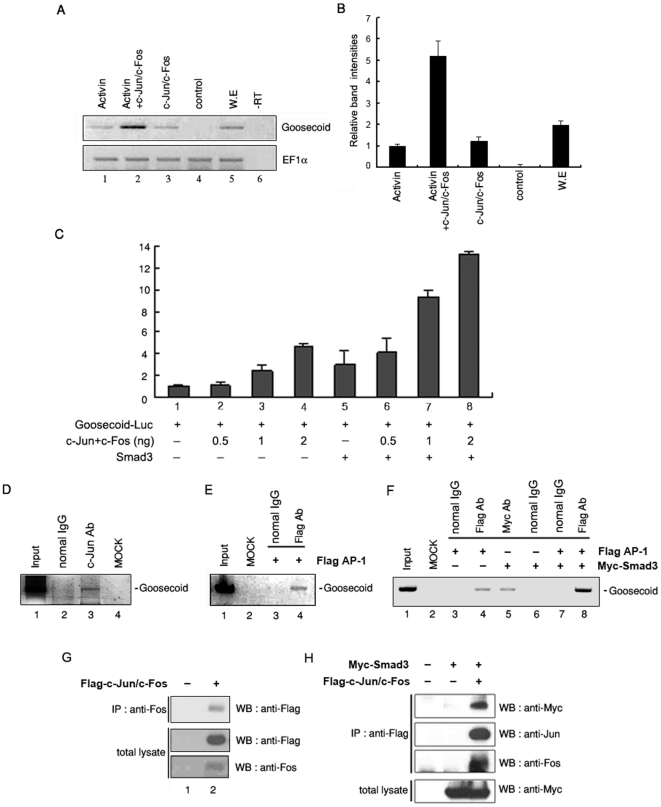
Heterodimeric AP-1^c-Jun/c-Fos^ directly regulates the transcription of goosecoid by physically interacting with Smad3, a mediator of activin signaling. (A) RT-PCR analysis of organizer genes in indicating mRNA injected animal caps explants (at stage 10.5 equivalent); lane 1, animal caps treated with activin (5 ng/ml); animal caps injected with 0.5 ng of AP-1^c-Jun/c-Fos^ mRNA were incubated in the absence (lane 3) or presence (lane2) of activin (5 ng/ml). Activin and AP-1 have synergistic effect on *goosecoid* expression. (B) Relative band intensities of A shown in panel. Relative band intensities were analyzed by ImageJ software provided by NIH, USA. (C) Luciferase assay using animal caps (at stage 10.5 equivalent) expressing *goosecoid* promoter luciferase reporter genes with the indicated mRNAs. All values are averages of at least three independent experiments. Smad3 and AP-1 have synergistic effect on enhancement of the *goosecoid* promoter activity. (D–F) Chromatin immunoprecipitation (ChIP) assay from whole embryos (D) or embryos injected with AP-1 (flag-tagged rat *c-ju*n and rat *c-fos* mRNAs) alone or together with Smad3 mRNAs (E & F). The presence of *goosecoid* promoter was detected by PCR using DNA samples obtained; after c-Jun antibody precipitation (D), Flag or Myc antibody precipitation (Flag Ab or Myc Ab) (E & F), IgG precipitation (normal IgG), and from cross-linked chromatin supernatant before immunoprecipitation (Input). MOCK was used as a control for the absence of DNA. (D) Endogenous c-Jun binds to the *goosecoid* promoter (lane 3). (E) Exogenous AP-1 binds to the *goosecoid* promoter (lane 4). (F) AP-1 and Smad3 cooperatively bind to *goosecoid* promoter (lane 8). (G & H) Immunoprecipitation assay from whole embryos injected with AP-1 (flag-tagged rat *c-ju*n and rat *c-fos* mRNAs) alone or together with Smad3 mRNAs (at stage 10.5 equivalent). Cell extracts from embryos used in ChIP assay were co-immunoprecipitated (IP) with the indicated antibodies. (G) The physical interaction of c-Jun and c-Fos in embryo. (H) The physical interaction of AP-1 and Smad3 in embryo.

To examine the cooperative role of AP-1^c-Jun/c-Fos^ and activin signaling, we checked the expression of *goosecoid* gene in animal cap explants. Using RT-PCR, we tested whether activin and AP-1^c-Jun/c-Fos^ have a synergistic effect on *goosecoid* expression which is the most well-known organizer directly regulated by activin signaling [29], [37]. Data revealed that the *goosecoid* mRNA levels were notably increased by co-treatment of activin and AP-1^c-Jun/c-Fos^ (Fig. 6A & B).

In order to verify whether this enhanced up-regulation of *goosecoid* mRNA levels by co-treatment of activin and AP-1^c-Jun/c-Fos^ are resulted from a cooperative interaction of Smad3 and AP-1^c-Jun/c-Fos^, we tested *gooscoid* promoter activity which contains activin response elements along with putative Smad binding sites. The reporter activity of *goosecoid* promoter containing 240 nucleotides from transcription start site (−240 bp *gooscoid*-Luc) was increased by AP-1^c-Jun/c-Fos^ in a dose dependent manner (Fig. 6C). When Smad3 was co-injected, the *goosecoid* promoter activity was synergistically enhanced at various doses of AP-1^c-Jun/c-Fos^ (Fig. 6C).

Based on these results, we examined whether AP-1^c-Jun/c-Fos^ and Smad3 proteins directly bind to the promoter region of *goosecoid*. To address this point, we examined physical binding of AP-1^c-Jun/c-Fos^ on the endogenous *goosecoid* promoter with endogenous c-Jun proteins by using ChIP assays. As shown in Fig. 5D, the 290 bp (−240/+50) of goosecoid promoter fragment harboring activin response element was detected from chromatin precipitated with endogenous c-Jun antibody. We also tested physical binding of AP-1^c-Jun/c-Fos^ on the endogenous *goosecoid* promoter with exogenous flag tagged c-Jun and Fos proteins by using ChIP assays. Consistent with Fig. 5D, exogenous AP-1^c-Jun/c-Fos^ was able to directly bind to goosecoid promoter (Fig. 5E) indicating that AP-1^c-Jun/c-Fos^ directly bind to the promoter region of *goosecoid in vivo*.

Furthermore, we tested incorporation of physically interacted Smad3 and AP-1 on *goosecoid* promoter. AP-1^c-Jun/c-Fos^ or Smad3 alone weakly binds to the *goosecoid* promoter region between −240 to +50 bp (Fig. 6F, lane 4 and 5). Interestingly, co-immunoprecipitation of AP-1^c-Jun/c-Fos^ and Smad3 with anti-Flag resulted in enhanced their binding activity on *goosecoid* promoter (Fig. 6F, lane 8). Equal amount of gene expression was confirmed by western blot (Supplementary Fig. S3). Our co-immunoprecipitation assay also confirmed a physical interaction between AP-1^c-Jun/c-Fo^ and Smad3 as well as c-Jun and c-Fos using cell extracts from *Xenopus* embryos used in ChIP assay (Figs. 6G & H). Taken together, our results suggest that physical interaction between heterodimeric AP-1^c-Jun/c-Fos^ and Smad3 synergistically activate transcription of *goosecoid* gene through direct binding to its promoter.

### Physical interaction between Smad3 and c-Jun is necessary for up-regulation of organizer genes

Previously, Jing Qing et al. characterized the functional cooperative site between c-Jun and Smad3. Especially, the replacement of Smad3's four lysines at positions 40, 41, 43 and 44 to alanines completely abolished its association with c-Jun [36].

**Figure 7:**
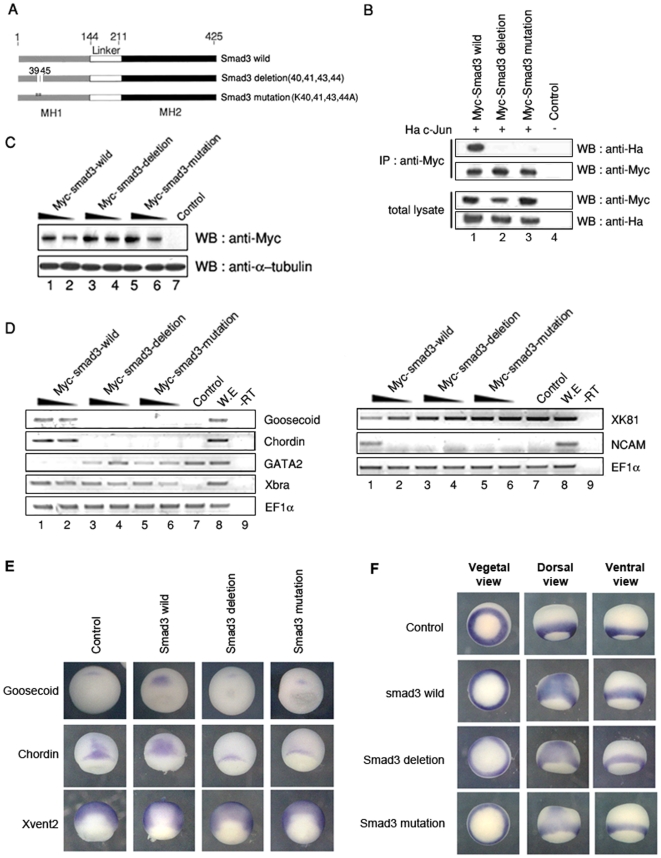
Physical interaction of Smad3 and AP-1 is necessary for full activation of organizer genes, but not for *Xbra*. (A) Schematic representation of Smad3 constructs used in this study. (B) Immunoprecipitation assay of c-Jun and wild Smad3 or Smad3 mutants. Co-immunoprecipitation of Smad3 mutants and c-Jun was not seen (lane 2, 3). (C–F) Whole embryos injected with **i**ndicated mRNAs or isolated animal caps were cultured until stage 10.5 and stage 24. (C) Embryos injected with indicated mRNA were lysised and performed western blot with anti-Myc for confirmation of mRNA expression. Injected mRNA of Smad3 mutants and wild type was translated into proteins in a dose-dependent manner. (D) RT-PCR on animal caps expressing indicated mRNAs. Unlike wild Smad3 (lane 1–2), Smad3 mutants lost inducing activity of organizer genes and neural specific marker (lane 3–6): EF-1α, a loading control; -rt, control reaction without reverse transcriptase; cont, animal caps sample dissected from non-injected embryos; we, whole embryo as a positive control; organizer gene, *chordin* and *goosecoid*; pan-neural marker, *N-CAM*; ventral mesoderm marker, *GATA2*; epidermis marker (non-neural marker), *XK81*; pan-mesoderm marker, *Xbra*. (E) Embryos injected with indicated mRNAs (upper panel) were processed for whole mount *in situ* hybridization with indicated markers (left panel). (F) Embryos injected with indicated mRNAs (left panel) were processed for whole mount *in situ* hybridization with *Xbra*.

To verify our hypothesis that physical interaction between Smad3 and AP-1 might be crucial to regulate *Xenopus* organizer's expression, we generated two Smad3 mutant constructs harboring deletions or point mutations on c-Jun binding site as shown in Fig. 7A [36].

Our immunoprecipitation assay revealed that wild-type Smad3 interacted with c-Jun, whereas both mutant types of Smad3 failed to interact with c-Jun (Fig. 7B).

We then assessed physiological significance by analyzing expression of organizer genes using RT-PCR. Dose-dependent expression of injected wild or mutant Smad3 mRNA was confirmed by western blot (Fig. 7C). Crucially, both mutant types of Smad3 completely lost their activity regulating the expression of organizer genes (*chordin* and *gooscoid*) as well as neural marker (*NCAM*) compared with wild-type Smad3 (Fig. 7D). However, expression of a pan-mesoderm marker *Xbra* was not affected, indicating that interaction of Smad3 with c-Jun is necessary for the expression of organizer genes, but not for *Xbra* (Fig. 7D). To verify these observations, we analyzed embryos injected with wild Smad3 or Smad3 mutants by whole mount in situ hybridization. Wild Smad3 injected embryos showed enhanced expression of *goosecoid* and *chordin*, whereas mutant Smad3 injected embryos showed reduced or similar expression of the genes compared with those of control embryos (Fig. 7E). Consistent with RT-PCR results (Fig. 7D), *Xbra* expression was enhanced either by wild-type and mutant Smad3, and the enhanced expression level was not affected by mutations on c-Jun binding site (Fig. 7F). Taken together, we concluded that physical interaction of Smad3 and AP-1 is necessary for the expression of organizer genes during *Xenopus* embryogenesis.

## Discussion

In the present study, we provide new evidence for a novel function of transcription factor AP-1^c-Jun/c-Fos^ in activin signaling during *Xenopus* early embryogenesis. Moreover, we demonstrate physiological function of physical interaction between AP-1/c-Jun and Smad3, suggesting functional specificity for AP-1^c-Jun/c-Fos^ in regulation of Spemann organizer genes. Previously, AP-1 has been shown to be involved in BMP signaling in *Xenopus* development [24]. Since AP-1 is a transcription factor that exerts a variety of cellular effects, a question regarding the functional specificity of AP-1 in activin signaling could be raised. Therefore, we here showed that heterodimeric AP-1^c-Jun/c-Fos^ induced the expression of dorsal mesoderm markers, namely BMP-antagonizing genes, whereas inhibited the expressions of a ventral (*PV.1*) and an epidermal marker (*XK81*) in a dose-dependent manner (Supplementary Fig. S1). Our reporter assays also verified that AP-1^c-Jun/c-Fos^ specifically enhanced the promoter activity of activin-response genes (Figs. 2). Moreover, overexpression of AP-1^c-Jun/c-Fos^ into ventral blastomeres partially changed ventrally-fated tissue into dorsally-fated tissue in whole embryo (Supplementary Fig. S4), indicating that AP-1^c-Jun/c-Fos^ functions similar to activin, but not like BMP.

We have reported that AP-1 is involved in mesoderm induction as a downstream mediator of FGF signaling [21] and that AP-1^c-Jun/c-Fos^ induces pan-mesodermal marker, *Xbra* [22]. For mesoderm maintenance, *Xbra* and embryonic FGF (eFGF) have been shown to require each other in the mode of autocatalytic loop. Notably, AP-1 has been shown to play an important role in this autocatalytic loop during mesoderm induction and maintenance [22]. We previously showed that AP-1-mediated *Xbra* expression is abolished by a dominant-negative mutant of the FGF receptor (DNFR) [22]. Unlike Xbra expression, however, organizer gene expression is not induced by any dose of FGF (eFGF and bFGF). This indicates that AP-1^c-Jun/c-Fos^ has a specific role in organizer gene expression that is distinct from FGF signaling.

A detail analysis of the *gooscoid* promoter region and of the interactions between Smad and AP-1 components would be interesting subjects in terms of understanding the relation between activin signaling and AP-1. We showed that AP-1^c-Jun/c-Fos^ or Smad3 are able to bind to the -240 *goosecoid* promoter region by using ChIP assays (Fig. 6F). To verify the specificity for the binding of Smad3 or AP-1 on *goosecoid* promoter, we tested the binding of these proteins into other region of *goosecoid*. Inability of Smad3 or AP-1 for the binding on the other region of *goosecoid* suggests the specificity of their binding on −240 *goosecoid* promoter region (Supplementary Fig. S5). Moreover, this interaction was enhanced in the presence of AP-1^c-Jun/c-Fos^ and Smad3 together (Fig. 6F). As described in Materials and Methods, −240 *goosecoid* promoter contains activin response elements of the *goosecoid* gene as named the distal element (DE). Interestingly, we found that the DE region contains the expected consensus AP-1 binding site. These results are consistent with synergistic effects between AP-1^c-Jun/c-Fos^ and Smad3 for the expression of *goosecoid* (Fig. 6A) or luciferase activity of goosecoid promoter (Fig. 6C). Collectively, these results provide evidence for direct regulation of organizer gene, *goosecoid*, transcription by AP-1^c-Jun/c-Fos^ via physical interaction with Smad3, a downstream mediator of activin signaling.

Additionally, we demonstrated functional specificity of AP-1^c-Jun/c-Fos^ in expression of organizer genes via interaction with a specific factor, Smad3. Although the interaction between Smad3 and AP-1^c-Jun/c-Fos^ has been previously reported in a mammalian cell line, the physiological function of this interaction has not been investigated in detail. In present study, we showed that physical interaction of Smad3 and AP-1^c-Jun/c-Fos^ provided specific role in their physiological function. Interestingly, we discriminate AP-1^c-Jun/c-Fos^-mediated *Xbra* expression and AP-1^c-Jun/c-Fos^-mediated organizer gene expression through Smad3 mutant experiment (Figs. 7). Wild-type Smad3 induced expression of *Xbra* and organizer genes like activin in animal cap explants. On the other hand, Smad3 mutants containing deletion and point mutation on c-Jun binding site did not induce expression of organizer genes. Interestingly, mutant Smad3 still maintained expression activity of *Xbra* gene (Fig. 7D), indicating that Smad3 requires AP-1/c-Jun binding only for organizer gene expressions but not for *Xbra*. Also depletion of c-Jun in DMZ (Fig. 4B) or in animal caps derived from Smad3-injected embryos (data not shown) did not significantly affect expression of *Xbra*. Therefore, our data shown in this study provided the first evidence for the specific involvement of AP-1 in the expression of Spemann organizer genes, not *Xbra*.

In this paper, our studies focused on the interaction between AP-1 and Smad3, but not Smad2 which is more likely to be the actual activin-like signaling in organizer formation [38], [39], [40]. Recent report also suggests that Smad3 proved to be a more powerful inducer of chordin than Smad2 in a Smicl-dependent mechanism [35]. Both Smad3 and Smad2 are able to induce expression of Spemann organizer genes in isolated *Xenopus* animal caps (data not shown). In this regard, the physiological relevance of interaction between Smad2 and AP-1 during the stages of organizer gene expression remains to be investigated. In this paper, we demonstrated for the first time that a specific combination of c-Jun and c-Fos plays an important role in activin signaling and BMP-antagonizing organizer gene expression, thereby it serves as an essential component of transcriptional machinery in early *Xenopus* embryogenesis. Moreover, we showed a physical interaction between AP-1^c-Jun/c-Fos^ and Smad3 is essential for expression of BMP-antagonizing organizer genes. Taken together, we suggest functional specificity of heterodimeric AP-1^c-Jun/c-Fos^ in induction of BMP-antagonizing organizer genes as a downstream component of activin signaling during *Xenopus* development.

## Supporting Information

Figure S1 **AP-1^c-Jun/c-Fos^ inhibited the expressions of PV.1 and XK81 (BMP-4 responsive genes) in dose dependent manner.** (**A and B**). Embryos were injected with the indicated concentrations of mRNAs encoding *c-jun* and *c-fos*. Animal caps were then isolated and cultured until stage 11 or 24. The expressions of ventral mesoderm marker (*PV.1*) and epidermis marker (*XK81*) were investigated by RT-PCR analysis. EF-1α, a loading control; -rt, control reaction without reverse transcriptase; cont, animal cap samples dissected from non-injected embryos; we, whole embryo as a positive control.
(TIF)

Figure S2 **A**, Embryos injected with 20 ng of Cont-MO were cultured until stage 10.5 and extracted protein was used for western blot with c-Jun and α-tubulin antibody. Cont-MO did not affect the expression of c-Jun protein. **B**, Animal caps isolated from embryos injected with Cont-MO or not were cultured in the presence or absence of activin (25 ng/ml). At stage 10.5, collected animal caps were used for RT-PCR analysis. Cont-MO did not affect Spemann organizer gene expression induced by activin.
(TIF)

Figure S3 **Injected mRNA of AP-1 and Smad3 was translated into proteins.** Expression of AP-1 and Smad3 was confirmed with Flag antibody and Myc antibody, respectively.
(TIF)

Figure S4 **AP-1^c-Jun/c-Fos^ converts ventral-fated tissue into dorsal tissue.** The 200 pg of individual GFP mRNA (**A**) or 500 pg of AP-1 (*c-jun* and *c-fos*) mRNA together (**B**) were injected into ventral-animal-blastomere**s** (V1 and V2) at the 8 cell stage and then cultured until stage 27–30. Embryos were fixed and GFP expressions were observed by Green Fluorescent Microscopy. Ventrally expressed GFP (circle region of A) was partially transferred into dorsal region (circle region of B).
(TIF)

Figure S5 **A**, Schematic representation of amplification region on goosecoid exon 3 for ChIP assay. **B**, Chromatins extracted from embryos injected with indicated myc-tagged smad3 mRNAs were immunoprecipitated with normal IgG or Myc antibody, respectively. Immunoprecipitated chromatin was used for PCR of goosecoid exon region. Arrow head indicated the amplification of goosecoid exon region. Control, non-injected embryo; No Chip, no antibody; Input, positive control of PCR.
(TIF)

## References

[1] Angel P, Karin M (1991). The role of Jun, Fos and the AP-1 complex in cell-proliferation and transformation.. Biochim Biophys Acta.

[2] Jochum W, Passegue E, Wagner EF (2001). AP-1 in mouse development and tumorigenesis.. Oncogene.

[3] Ameyar M, Wisniewska M, Weitzman JB (2003). A role for AP-1 in apoptosis: the case for and against.. Biochimie.

[4] Eferl R, Wagner EF (2003). AP-1: a double-edged sword in tumorigenesis.. Nat Rev Cancer.

[5] Shaulian E, Karin M (2002). AP-1 as a regulator of cell life and death.. Nat Cell Biol.

[6] Shaulian E, Karin M (2001). AP-1 in cell proliferation and survival.. Oncogene.

[7] Kessler DS, Melton DA (1994). Vertebrate embryonic induction: mesodermal and neural patterning.. Science.

[8] Kimelman D, Christian JL, Moon RT (1992). Synergistic principles of development: overlapping patterning systems in Xenopus mesoderm induction.. Development.

[9] Slack JM (1994). Inducing factors in Xenopus early embryos.. Curr Biol.

[10] Amaya E, Stein PA, Musci TJ, Kirschner MW (1993). FGF signalling in the early specification of mesoderm in Xenopus.. Development.

[11] Isaacs HV, Tannahill D, Slack JM (1992). Expression of a novel FGF in the Xenopus embryo. A new candidate inducing factor for mesoderm formation and anteroposterior specification.. Development.

[12] Kimelman D, Kirschner M (1987). Synergistic induction of mesoderm by FGF and TGF-beta and the identification of an mRNA coding for FGF in the early Xenopus embryo.. Cell.

[13] Hemmati-Brivanlou A, Melton DA (1992). A truncated activin receptor inhibits mesoderm induction and formation of axial structures in Xenopus embryos.. Nature.

[14] Smith JC, Price BM, Green JB, Weigel D, Herrmann BG (1991). Expression of a Xenopus homolog of Brachyury (T) is an immediate-early response to mesoderm induction.. Cell.

[15] Ruiz i Altaba A, Melton DA (1989). Interaction between peptide growth factors and homoeobox genes in the establishment of antero-posterior polarity in frog embryos.. Nature.

[16] Christian JL, McMahon JA, McMahon AP, Moon RT (1991). Xwnt-8, a Xenopus Wnt-1/int-1-related gene responsive to mesoderm-inducing growth factors, may play a role in ventral mesodermal patterning during embryogenesis.. Development.

[17] von Dassow G, Schmidt JE, Kimelman D (1993). Induction of the Xenopus organizer: expression and regulation of Xnot, a novel FGF and activin-regulated homeo box gene.. Genes Dev.

[18] Green JB, Smith JC (1990). Graded changes in dose of a Xenopus activin A homologue elicit stepwise transitions in embryonic cell fate.. Nature.

[19] Green JB, Howes G, Symes K, Cooke J, Smith JC (1990). The biological effects of XTC-MIF: quantitative comparison with Xenopus bFGF.. Development.

[20] Smith JC (1993). Mesoderm-inducing factors in early vertebrate development.. Embo J.

[21] Dong Z, Xu RH, Kim J, Zhan SN, Ma WY (1996). AP-1/jun is required for early Xenopus development and mediates mesoderm induction by fibroblast growth factor but not by activin.. J Biol Chem.

[22] Kim J, Lin JJ, Xu RH, Kung HF (1998). Mesoderm induction by heterodimeric AP-1 (c-Jun and c-Fos) and its involvement in mesoderm formation through the embryonic fibroblast growth factor/Xbra autocatalytic loop during the early development of Xenopus embryos.. J Biol Chem.

[23] Kim J, Ault KT, Chen HD, Xu RH, Roh DH (1998). Transcriptional regulation of BMP-4 in the Xenopus embryo: analysis of genomic BMP-4 and its promoter.. Biochem Biophys Res Commun.

[24] Xu RH, Dong Z, Maeno M, Kim J, Suzuki A (1996). Involvement of Ras/Raf/AP-1 in BMP-4 signaling during Xenopus embryonic development.. Proc Natl Acad Sci U S A.

[25] Smith JC, Slack JM (1983). Dorsalization and neural induction: properties of the organizer in Xenopus laevis.. J Embryol Exp Morphol.

[26] Ariizumi T, Takahashi S, Chan TC, Ito Y, Michiue T (2009). Isolation and differentiation of Xenopus animal cap cells.. Curr Protoc Stem Cell Biol Chapter 1: Unit 1D.

[27] Okabayashi K, Asashima M (2003). Tissue generation from amphibian animal caps.. Curr Opin Genet Dev.

[28] Tao QH, Yang J, Mei WY, Geng X, Ding XY (1999). Cloning and analysing of 5′ flanking region of Xenopus organizer gene noggin.. Cell Res.

[29] Watabe T, Kim S, Candia A, Rothbacher U, Hashimoto C (1995). Molecular mechanisms of Spemann's organizer formation: conserved growth factor synergy between Xenopus and mouse.. Genes Dev.

[30] Knochel S, Schuler-Metz A, Knochel W (2000). c-Jun (AP-1) activates BMP-4 transcription in Xenopus embryos.. Mech Dev.

[31] Summerton J, Weller D (1997). Morpholino antisense oligomers: design, preparation, and properties.. Antisense Nucleic Acid Drug Dev.

[32] Messenger NJ, Kabitschke C, Andrews R, Grimmer D, Nunez Miguel R (2005). Functional specificity of the Xenopus T-domain protein Brachyury is conferred by its ability to interact with Smad1.. Dev Cell.

[33] Moore KB, Mood K, Daar IO, Moody SA (2004). Morphogenetic movements underlying eye field formation require interactions between the FGF and ephrinB1 signaling pathways.. Dev Cell.

[34] Weinstein M, Yang X, Deng C (2000). Functions of mammalian Smad genes as revealed by targeted gene disruption in mice.. Cytokine Growth Factor Rev.

[35] Collart C, Verschueren K, Rana A, Smith JC, Huylebroeck D (2005). The novel Smad-interacting protein Smicl regulates Chordin expression in the Xenopus embryo.. Development.

[36] Qing J, Zhang Y, Derynck R (2000). Structural and functional characterization of the transforming growth factor-beta -induced Smad3/c-Jun transcriptional cooperativity.. J Biol Chem.

[37] Cho KW, Blumberg B, Steinbeisser H, De Robertis EM (1991). Molecular nature of Spemann's organizer: the role of the Xenopus homeobox gene goosecoid.. Cell.

[38] Grimm OH, Gurdon JB (2002). Nuclear exclusion of Smad2 is a mechanism leading to loss of competence.. Nat Cell Biol.

[39] Howell M, Mohun TJ, Hill CS (2001). Xenopus Smad3 is specifically expressed in the chordoneural hinge, notochord and in the endocardium of the developing heart.. Mech Dev.

[40] Faure S, Lee MA, Keller T, ten Dijke P, Whitman M (2000). Endogenous patterns of TGFbeta superfamily signaling during early Xenopus development.. Development.

