# Supplementary figures and images for: The Function of Heterodimeric AP-1 Comprised of c-Jun and c-Fos in Activin Mediated Spemann Organizer Gene Expression
Source: PLoS One. 2011 Jul 29;6(7):e21796. doi: 10.1371/journal.pone.0021796 (PMC3146467; doi:10.1371/journal.pone.0021796)

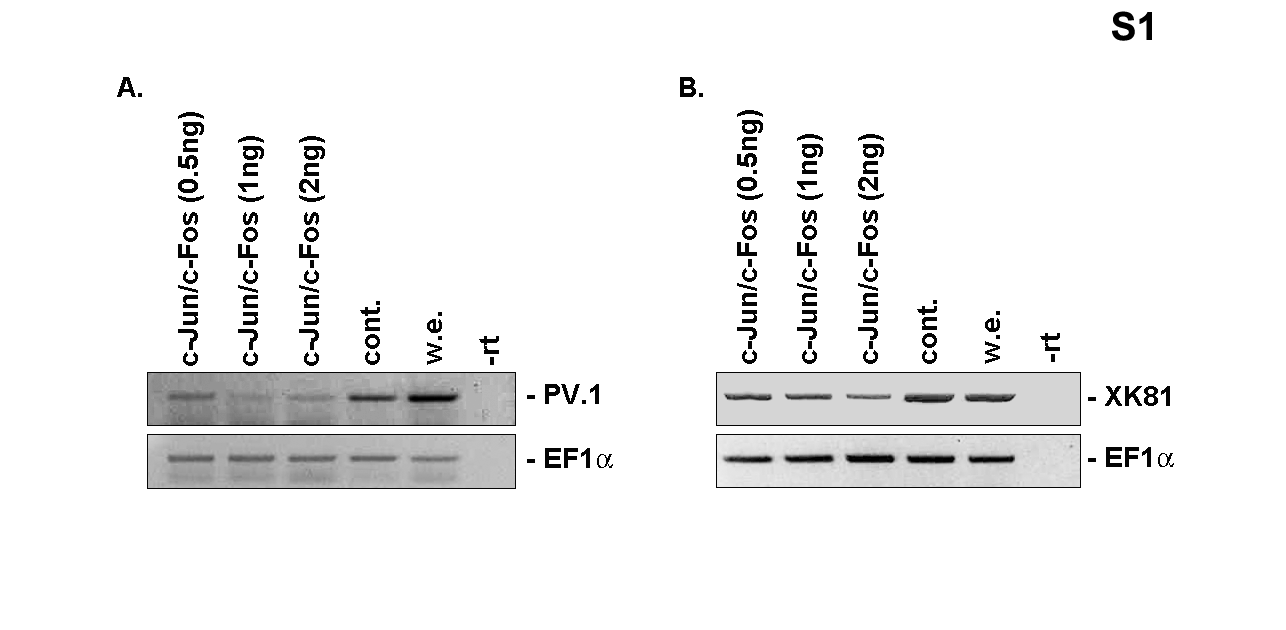

Supplement: Figure S1 — AP-1c-Jun/c-Fos inhibited the expressions of PV.1 and XK81 (BMP-4 responsive genes) in dose dependent manner. (A and B). Embryos were injected with the indicated concentrations of mRNAs encoding c-jun and c-fos. Animal caps were then isolated and cultured until stage 11 or 24. The expressions of ventral mesoderm marker (PV.1) and epidermis marker (XK81) were investigated by RT-PCR analysis. EF-1α, a loading control; -rt, control reaction without reverse transcriptase; cont, animal cap samples dissected from non-injected embryos; we, whole embryo as a positive control. (TIF) [file pone.0021796.s001.tif]

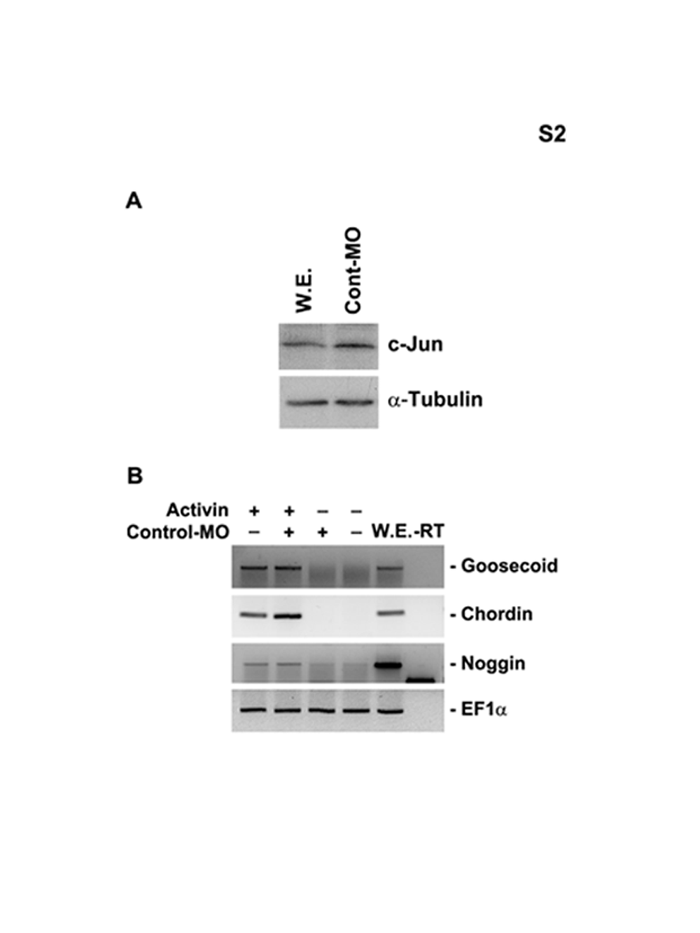

Supplement: Figure S2 — A, Embryos injected with 20 ng of Cont-MO were cultured until stage 10.5 and extracted protein was used for western blot with c-Jun and α-tubulin antibody. Cont-MO did not affect the expression of c-Jun protein. B, Animal caps isolated from embryos injected with Cont-MO or not were cultured in the presence or absence of activin (25 ng/ml). At stage 10.5, collected animal caps were used for RT-PCR analysis. Cont-MO did not affect Spemann organizer gene expression induced by activin. (TIF) [file pone.0021796.s002.tif]

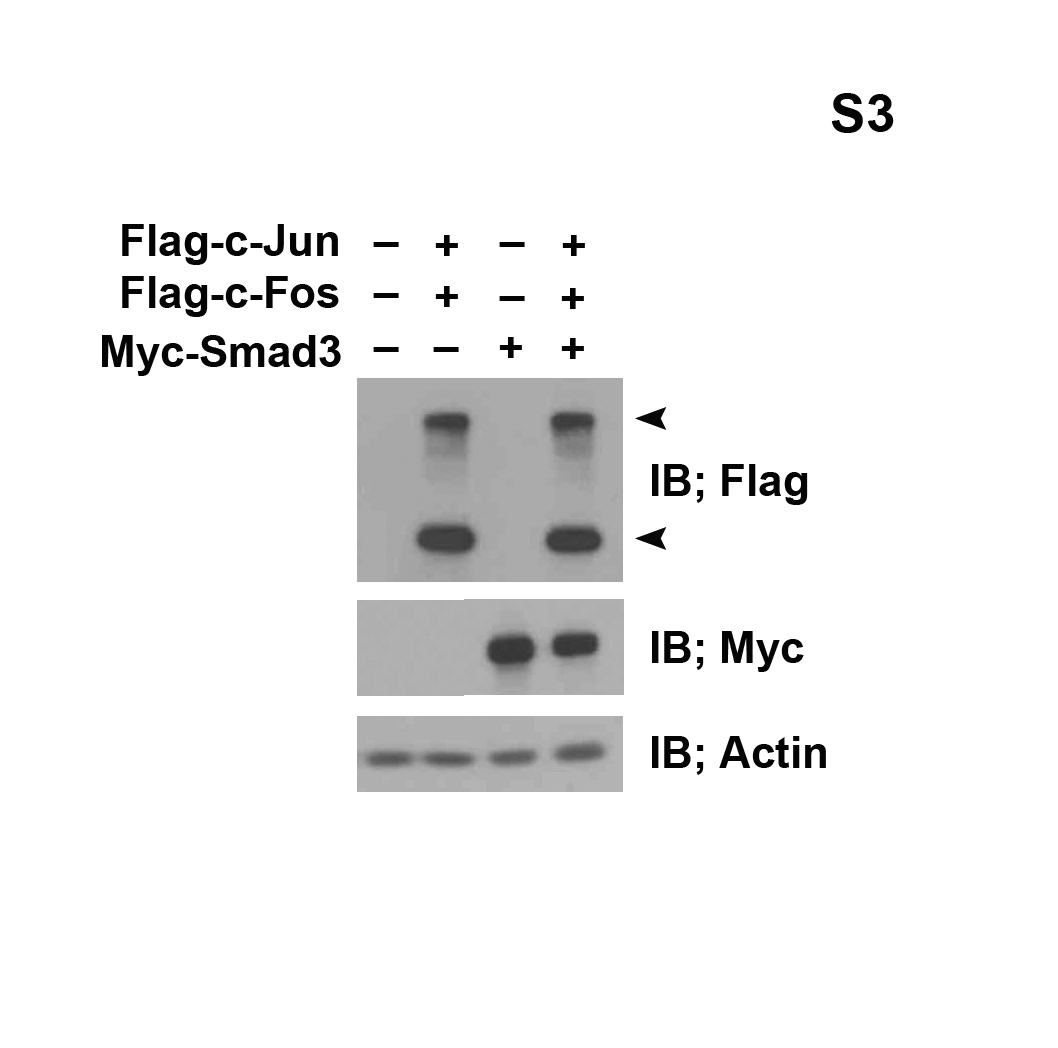

Supplement: Figure S3 — Injected mRNA of AP-1 and Smad3 was translated into proteins. Expression of AP-1 and Smad3 was confirmed with Flag antibody and Myc antibody, respectively. (TIF) [file pone.0021796.s003.tif]

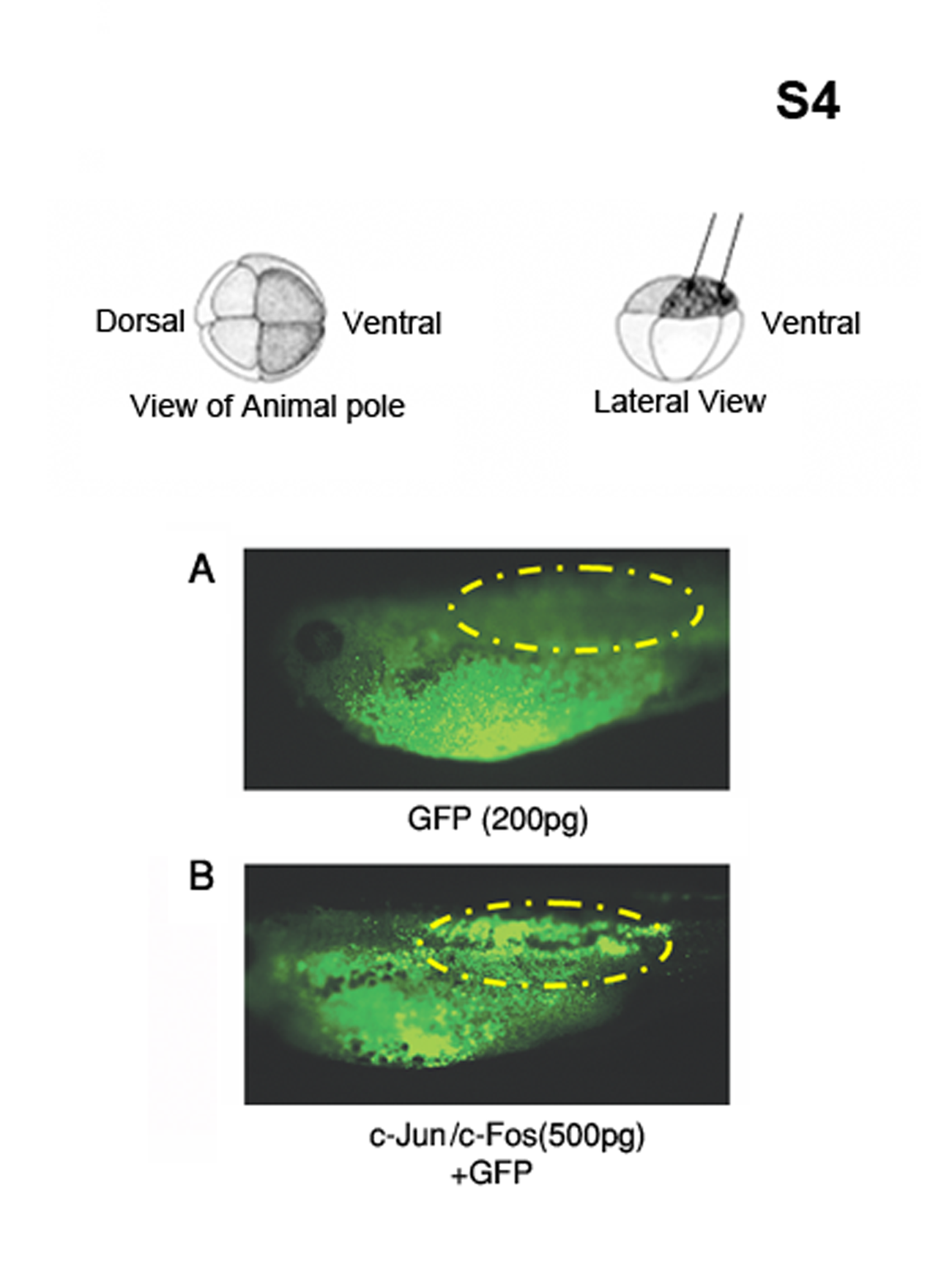

Supplement: Figure S4 — AP-1c-Jun/c-Fos converts ventral-fated tissue into dorsal tissue. The 200 pg of individual GFP mRNA (A) or 500 pg of AP-1 (c-jun and c-fos) mRNA together (B) were injected into ventral-animal-blastomeres (V1 and V2) at the 8 cell stage and then cultured until stage 27–30. Embryos were fixed and GFP expressions were observed by Green Fluorescent Microscopy. Ventrally expressed GFP (circle region of A) was partially transferred into dorsal region (circle region of B). (TIF) [file pone.0021796.s004.tif]

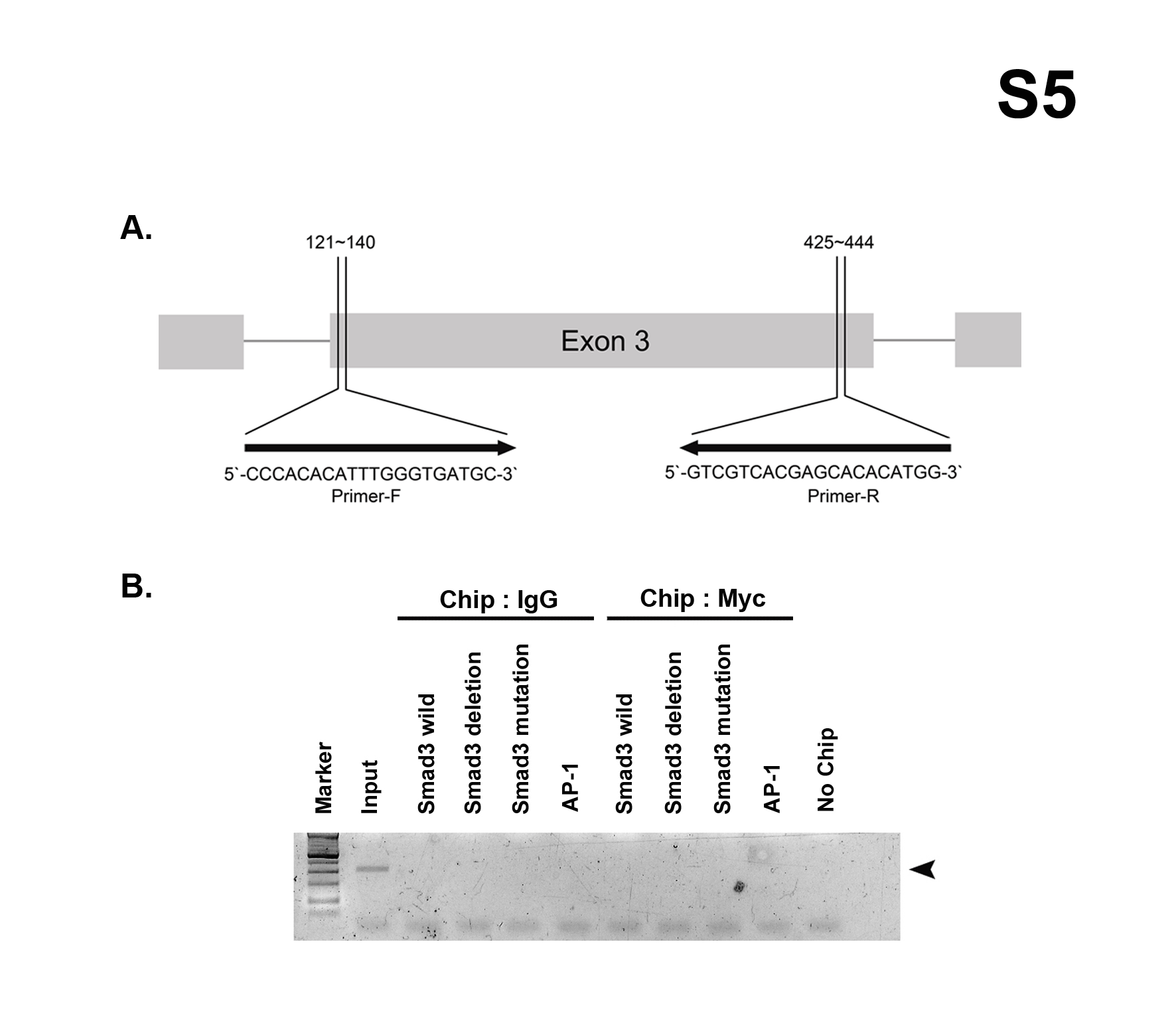

Supplement: Figure S5 — A, Schematic representation of amplification region on goosecoid exon 3 for ChIP assay. B, Chromatins extracted from embryos injected with indicated myc-tagged smad3 mRNAs were immunoprecipitated with normal IgG or Myc antibody, respectively. Immunoprecipitated chromatin was used for PCR of goosecoid exon region. Arrow head indicated the amplification of goosecoid exon region. Control, non-injected embryo; No Chip, no antibody; Input, positive control of PCR. (TIF) [file pone.0021796.s005.tif]
